# SEOM clinical guidelines in early stage breast cancer (2018)

**DOI:** 10.1007/s12094-018-1973-6

**Published:** 2018-11-15

**Authors:** F. Ayala de la Peña, R. Andrés, J. A. Garcia-Sáenz, L. Manso, M. Margelí, E. Dalmau, S. Pernas, A. Prat, S. Servitja, E. Ciruelos

**Affiliations:** 1 Department of Hematology and Medical Oncology, Hospital G. Universitario Morales Meseguer, Avda. Marqués de los Vélez, s/n, 30001 Murcia, Spain; 2 Division of Medical Oncology, Hospital Clínico Universitario Lozano Blesa, Zaragoza, Spain; 3 Department of Medical Oncology, Hospital Clínico Universitario San Carlos, Madrid, Spain; 4 Department of Medical Oncology, University Hospital, 12 de Octubre, Madrid, Spain; 5 Department of Medical Oncology, Breast Cancer Unit, B-ARGO Group, Institut Català d’Oncologia, Badalona, Spain; 6 Department of Oncology, Parc Taulí Sabadell, Hospital Universitari, Barcelona, Spain; 7 Department of Medical Oncology, Breast Cancer Unit, Institut Català d’Oncologia, Barcelona, Spain; 8 Department of Medical Oncology, Hospital Clínic, Barcelona, Spain; 9 Department of Medical Oncology, Hospital del Mar, Barcelona, Spain; 10 Department of Medical Oncology, Breast Cancer Unit, University Hospital, 12 de Octubre, Madrid, Spain; 11 HM Hospitales, Madrid, Spain

**Keywords:** Early breast cancer, Adjuvant, Neoadjuvant, Genomic predictive test, Follow-up

## Abstract

Breast cancer is the most common cancer in women in our country and it is usually diagnosed in the early and potentially curable stages. Nevertheless, around 20–30% of patients will relapse despite appropriate locoregional and systemic therapies. A better knowledge of this disease is improving our ability to select the most appropriate therapy for each patient with a recent diagnosis of an early stage breast cancer, minimizing unnecessary toxicities and improving long-term efficacy.

## Introduction

Breast cancer is a major public health problem due to its high incidence, prevalence, and mortality. It is by far the most common cancer among women in Spain (2012), accounting for 29% of all new cases of cancer in females. Moreover, it is the first cause of cancer-related mortality in the female population, accounting for 15.5% of female cancer deaths, and the 5-year prevalence is 40.8% [1].

Breast cancer is a heterogeneous disease with multiple intrinsic tumor subtypes [2]. Up to one in three patients will develop metastases depending on lymph node involvement and breast cancer subtype, despite remarkable progress in early diagnosis and treatment. A better understanding of prognostic and predictive factors is allowing individualization of treatment for early stage breast cancer patients. The aim of these guidelines is to summarize current evidence and to give evidence-based recommendations for clinical practice [3].

## Methodology

These SEOM Guidelines have been developed with the consensus of ten breast cancer medical oncologists from the cooperative groups GEICAM (Spanish Breast Cancer Research Group) and SOLTI (Spanish Collaborative Group for the Study, Treatment and Other Experimental Strategies in Solid Tumors). To assign a level of evidence and a grade of recommendation to the different statements of this treatment guideline, it was decided to use the Infectious Diseases Society of America-US Public Health Service Grading System for Ranking Recommendations in Clinical Guidelines to determine the quality of evidence and strength of recommendation in each of the consensus recommendations (Table 1). A full list of recommendations is provided in Table 2.

**Table 1:** Strength of recommendation and quality of evidence score

Category, grade	Definition
Strength of recommendation	
A	Good evidence to support a recommendation for use
B	Moderate evidence to support a recommendation for use
C	Poor evidence to support a recommendation
D	Moderate evidence to support a recommendation against use
E	Good evidence to support a recommendation against use
Quality of evidence	
I	Evidence from ≥ 1 properly randomized, controlled trial
II	Evidence from ≥ 1 well-designed clinical trial, without randomization; from cohort or case-controlled analytic studies (preferably from > 1 center); from multiple time series; or from dramatic results from uncontrolled experiments
III	Evidence from opinions of respected authorities, based on clinical experience, descriptive studies, or reports of expert committees

**Table 2:** SEOM clinical practice guidelines for early breast cancer (2018 update): summary of recommendations

Recommendation	Category, grade
Diagnosis and initial workup	
Bilateral mammography and ultrasound of breast and regional lymph nodes in patients with suspected breast cancer	I, A
Core needle biopsy including evaluation of estrogen and progesterone receptors together with HER2 expression in patients with suspected breast cancer	I, A
Fine needle aspiration or core biopsy of suspicious lymph nodes	II, A
Bilateral breast MRI, with histologic confirmation of additional findings, as part of initial staging in patients with confirmed breast cancer, especially in patients with positive axillary nodes and occult primary breast cancer, Paget´s disease of the nipple, lobular carcinoma and multifocal or multicentric lesions	I, B
Laboratory test as part of initial staging of patients with confirmed breast cancer	III, B
Additional staging with chest and abdomen CT and bone scan in patients with stage III disease and/or with clinical or laboratory suggesting metastases	III, B
Additional staging with PET-CT in patients with locally advanced breast cancer	II, A
Evaluation of cardiac function in patients requiring anthracyclines and/or trastuzumab	I, A
Surgery	
Consideration of breast-conserving surgery as first surgical option in stages I–II	I, A
No indication of additional excision in patients with no ink on invasive tumor or DCIS after breast-conserving surgery	I, A
Contralateral mastectomy should be offered through an appropriate counseling process in BRCA1/2 mutation carriers	I, A
Sentinel lymph node (SLN) biopsy in patients with clinically negative axillary nodes	I, A
Omission of axillary lymph node dissection in patients with stage I–II disease and less than three positive axillary nodes after SLN biopsy and lumpectomy followed by adjuvant systemic therapy and radiotherapy	I, A
In patients with cN0 tumors, additional treatment of axilla is not necessary if SLNB is negative after neoadjuvant chemotherapy	I, A
In patients with cN + tumors, if SLNB is negative after neoadjuvant chemotherapy (NAC):	
ALND should be considered the standard procedure	I, A
ALND may be omitted in selected cases (pre-treatment marking of involved node, recovery of at least 2 SLNs, double-tracer technique)	II, C
Adjuvant radiotherapy	
Hypofractionated schemes are preferred for external beam whole radiation therapy after breast-conserving surgery	I, A
In elderly patients, the benefit of radiotherapy after breast-conserving surgery for stage I tumors should be assessed individually	I, A
Nodal (supra-and infra-clavicular) irradiation should be administered in patients with four or more involved nodes after breast-conserving surgery	I, A
Nodal (supra-and infra-clavicular) irradiation is recommended in patients with one to three involved nodes after breast-conserving surgery	I, B
In cN0 patients with pT1-2 tumors and positive SLNB, axillary irradiation may be used as an alternative to ALND	I, B
Chest wall and regional node (supraclavicular and internal mammary) irradiation should be administered in patients with four or more involved nodes, T4 or T3N+ tumors after mastectomy	I, A
Chest wall irradiation is recommended in patients with close or positive margins after mastectomy	II, A
Post-mastectomy irradiation should be considered in patients with T1-2 tumors and one to three involved nodes, or in patients with T3N0 tumors, after individually balancing risk factors for recurrence and patient preferences	I, B
Adjuvant radiotherapy is recommended after NAC in patients with stage III and T3N0 disease, regardless of response to NAC, and in patients with node-positive disease after NACT	I, A
Adjuvant radiotherapy may be omitted after NAC in patients with stage II (excluding T3N0) who achieve a pCR and do not have additional risk factors for recurrence	I, B
Decision-making for systemic adjuvant treatment	
OncotypeDX recurrence score (RS) may be used for prediction of the risk of distant recurrence at 9 years in patients with hormone receptor (HR)-positive and HER2-negative breast cancer treated with adjuvant endocrine therapy-only	I, A
OncotypeDX recurrence score (RS) may be used for prediction of the benefit of adjuvant chemotherapy in patients with hormone receptor (HR)-positive and HER2-negative	I, A
Prosigna risk of distant recurrence score (ROR) may be used for prediction of the risk of distant recurrence at 10 years in patients with HR-positive and HER2-negative breast cancer treated with adjuvant endocrine therapy-only for 5 years	I, B
Prosigna risk of distant recurrence score (ROR) may be used for prediction of the risk of late distant recurrence (years 5–10) in patients with HR-positive and HER2-negative breast cancer treated with adjuvant endocrine therapy-only for 5 years	I, B
Mammaprint risk score may be used for prediction of the risk of distant recurrence at 5 years in patients with hormone receptor (HR)-positive and HER2-negative breast cancer treated with adjuvant endocrine therapy-only	I, A
EndoPredict Epclin score may be used for prediction of the risk of distant recurrence at 10 years in patients with HR-positive and HER2-negative breast cancer treated with adjuvant endocrine therapy-only for 5 years	I, B
EndoPredict Epclin score may be used for prediction of the risk of late distant recurrence (years 5–10) in patients with HR-positive and HER2-negative breast cancer treated with adjuvant endocrine therapy-only for 5 years	I, B
Adjuvant and neoadjuvant systemic treatment of luminal breast cancer	
Adjuvant endocrine therapy (ET) should be offered to any patient with HR-positive breast cancer	I, A
Adjuvant ET with tamoxifen for 5 years is recommended as a standard treatment for premenopausal women with HR-positive breast cancer	I, A
Extended adjuvant ET with tamoxifen up to 10 years should be considered in high-risk patients	I, B
Adjuvant ET with exemestane plus ovarian function suppression should be offered to high-risk premenopausal breast cancer patients treated with adjuvant chemotherapy or in very young (under 35 years) women.	I, A
In high-risk patients not suitable or intolerant to aromatase inhibitors, tamoxifen plus ovarian function suppression may be considered as an alternative	II, B
Adjuvant ET for postmenopausal patients may consist in any of the following alternatives, after considering risk factors and individual preferences:	
Aromatase inhibitor (AI) during 5 years	I, A
Tamoxifen for 2–3 years followed by an AI to complete 5 years	I, A
Tamoxifen for 2–3 years followed by an AI during 5 years	II, B
Tamoxifen for 4.5–6 years followed by 2.5–5 years of an AI	I, A
Adjuvant treatment with oral or intravenous bisphosphonates should be considered in postmenopausal patients with breast cancer who are candidates for adjuvant systemic therapy	I, A
Adjuvant chemotherapy with standard anthracyclines and/or taxane regimens for luminal breast cancer is recommended for tumors defined by either clinical or genomic risk factors: T2-4, axillary node involvement N2-3, extensive LVI, high Ki67, low ER expression, younger age and premenopausal status, and intermediate to high genomic score.	I, A
NAC is indicated in locally advanced hormone receptor (HR)-positive and HER2-negative breast cancer	I, A
A sequential regimen of anthracyclines and taxanes is recommended in those patients in which NAC is indicated for hormone receptor (HR)-positive and HER2-negative breast cancer	II, B
Aromatase inhibitors are preferred over tamoxifen in postmenopausal patients in which neoadjuvant endocrine therapy is indicated for hormone receptor (HR)-positive and HER2-negative breast cancer	I, A
Neoadjuvant endocrine therapy with AI plus ovarian suppression or with tamoxifen might be considered in selected premenopausal patients in which chemotherapy is not an option	II, D
Adjuvant and neoadjuvant systemic treatment of HER2 breast cancer	
Addition of adjuvant trastuzumab to chemotherapy is recommended for HER2-positive breast cancer both in node-positive and in node-negative tumors with a tumor size > 1 cm	I, A
12-month duration of trastuzumab should be considered standard	I, A
Addition of adjuvant trastuzumab to chemotherapy should be considered in most cases of node-negative HER2-positive breast cancer with tumor size of 0.5–1.0 cm	II, B
For adjuvant chemotherapy of HER2-positive breast cancer, AC or EC for 4 cycles followed by 3 months of paclitaxel (P) or docetaxel (D or T) both in combination with trastuzumab (AC/EC → P/D + H) or docetaxel, carboplatin and trastuzumab (TCH) are the preferred regimens	I, A
In node-negative stage I tumors, an alternative less intense regimen with single-agent paclitaxel and trastuzumab for 12 weeks followed by single-agent trastuzumab (to complete a year) may be considered	II, B
Adjuvant dual HER2 blockade with trastuzumab and Pertuzumab for 18 cycles may be considered in patients with high-risk (node-positive and/or HR-negative) HER2-positive breast cancer. In patients that have received neoadjuvant treatment, Pertuzumab may be continued after surgery up to 18 cycles	I, B
Extended adjuvant treatment with neratinib after one year of trastuzumab may be considered in patients with node-positive and HR-positive HER2-positive breast cancer	I, B
Dual blockade with trastuzumab and pertuzumab and chemotherapy should be considered for the treatment of HER2-positive breast cancer patients who meet criteria for neoadjuvant treatment (> 2 cm tumor size and/or node-positive)	II, B
Adjuvant and neoadjuvant systemic treatment of triple-negative breast cancer	
Adjuvant chemotherapy for triple-negative breast cancer should include an anthracycline and a taxane, although the regimen docetaxel-cyclophosphamide might be considered in patients with a high risk for cardiac toxicity	I, B
No adjuvant chemotherapy is recommended for node-negative pT1a triple-negative breast cancer	III, B
Adjuvant chemotherapy may be considered for 0.6–1 cm tumors after an adequate individualized balance	III, B
Neoadjuvant chemotherapy for triple-negative breast cancer should include sequential anthracyclines and taxanes	I, A
Carboplatin may be considered as part of neoadjuvant chemotherapy for triple-negative breast cancer patients	I, A
Adjuvant capecitabine for 6-8 cycles should be considered in high-risk triple-negative breast cancer with residual invasive disease at surgery following standard neoadjuvant chemotherapy	I, B
Follow-up of early breast cancer	
Breast cancer surveillance is recommended in order to detect breast cancer recurrence and second primary tumors, assess physical and psychosocial long-term effects of breast cancer and its treatment, and promote a healthy lifestyle	I, A
Healthy lifestyles are recommended to prevent tumor recurrence	II, A
Early breast cancer follow-up should include regular visits every 3–6 months in the first 2 years, every 6 months from years 3–5 and annually thereafter	III, A
Annual ipsilateral (after breast-conserving surgery) and/or a contralateral mammography with ultrasound is recommended for follow-up of early breast cancer	II, A

## Diagnosis and initial workup

The following tests allow a correct diagnosis and prognosis approach to all patients in whom the presence of a breast tumor is suspected.

- Bilateral mammography and ultrasound of the breast and regional lymph nodes [I, A] [4]. Several new techniques such as 3D mammography or 3D ultrasound can increase diagnostic accuracy but are not routinely implemented.
- Core needle biopsy (preferably by ultrasound or stereotactic guidance). The study has to include the evaluation of the estrogen and progesterone receptor and HER2 gene expression [I, A]. Given the high inter-observer variability in the Ki-67 determination, it is important to be careful when using Ki67 to inform the decision-making process [5]. Fine needle aspiration or core biopsy of suspicious lymph nodes is recommended [II, A].
- MRI: is the most sensitive method for breast cancer staging but additional findings must be confirmed histologically due to the high false-positivity rate. Its use is not mandatory [I, B] and should be considered in cases of positive axillary nodes and occult primary breast cancer, Paget´s disease of the nipple, lobular carcinoma and multifocal or multicentric lesions. It is recommended prior and after neoadjuvant treatment to define the extent of disease and monitor the response to treatment [III, A] [6].
- Additional studies: anamnesis with personal and family medical history and complete physical examination. Lab test (complete blood count, liver and renal function, alkaline phosphatase and calcium) are routinely used but do not seem to improve detection of occult metastatic disease [III, B] [7].

When disease is detected in stage III or when signs or symptoms or laboratory values suggest suspected metastasis, a more extensive study with thoracic CT, abdominal CT and bone scan [III, B] should be performed. PET/CT is also recommended for initial staging in locally advanced BC (LABC) [II, A] [8].

Evaluation of cardiac function is imperative when using anthracyclines or trastuzumab [I, A] [9].

## Principles of surgery

Breast-conserving surgery is equivalent to mastectomy and must be considered as first option in most cases of stages I–II (I, A) [10]. No ink on invasive tumor or DCIS is considered an adequate margin after breast-conserving surgery [I, A] [11]. Mastectomy is still indicated in the following situations: tumor multicentricity, inability to achieve negative surgical margins after multiple resections, small breast size according to tumor volume, prior radiation therapy in the breast, or other contraindications for radiotherapy.

When mastectomy is performed, contralateral mastectomy as a prophylactic procedure is not indicated in most of the patients [12]. However, for carriers of BRCA1/2 mutations, the contralateral mastectomy should be offered through an appropriate risk–benefit assessment and counseling process [I, A] [13].

Sentinel lymph node biopsy (SLNB) is recommended for assessment of the involvement of axillary lymph nodes and should be performed in patients with clinically negative axillary nodes [I, A]. In patients with clinically positive axillary nodes, pathologic confirmation must be done by ultrasonography-guided fine needle aspiration (FNA) or core biopsy. Axillary lymph node dissection (ALND) is indicated in patients with positive SLN biopsy or confirmed preoperative pathologic axillary lymph node involvement. However, in patients with stage I-II disease and less than three positive axillary nodes after SLN biopsy and lumpectomy (and adjuvant radiotherapy indicated), ALND can be avoided without significant negative impact in DFS and OS when an adequate systemic postoperative treatment is provided [I, A] [14].

SLNB may be offered in patients with operable breast cancer before or after neoadjuvant chemotherapy (NACT). In patients with cN0 axilla, additional axillary treatment is not necessary if SLNB is negative after NACT [I, A] [15]. In patients with cN+ axilla who achieved ycN0 status after NACT, the ALND is recommended as standard procedure due to the high false-negative rate of the SLNB [I, A] [16]. In selected cN+ cases, in which positive axillary node has been marked prior to NACT, the identification and recovery of > 2 SLNs (including the marked node) with a double-contrast technique (Tc99 and methylene blue) may avoid ALND [II, C].

## Recommendations of adjuvant radiotherapy

Adjuvant radiotherapy (RT) should be performed in the case of:

- Breast-conserving surgery: external beam whole radiation therapy (WBRT). Hypofractionation schemes are preferred [I, A] [17]. If four or more nodes are involved, supra and infraclavicular radiotherapy is recommended [I, A]. In patients with one to three involved nodes after breast-conserving surgery, supra and infraclavicular nodal irradiation is recommended to minimize the risk of recurrence and potentially improve disease-specific survival [I, B] [18]. In addition, in patients with T1-2 tumors and cN0 and sentinel lymph node metastases, axillary irradiation is an alternative comparable to the ALND with less morbidity [I, B] [19].
- Mastectomy: chest wall and regional node irradiation including supraclavicular and internal mammary region is recommended in T4 tumors, node-positive T3 tumors, and if involvement of ≥ 4 axillary lymph nodes [I, A]. In cases of close or positive margins, chest wall irradiation is also recommended [II, A] [18, 20]. T1-2 tumors with one to three involved nodes and T3N0 tumors have an increased risk of locoregional recurrence after mastectomy. Post-mastectomy RT reduces the risk of recurrence and mortality and should be considered in these patients. However, benefit of adjuvant RT can be small in some subgroups of patients and must be discussed based on other risk factors such as grade, age, lymphovascular invasion, receptor status or lack of systemic therapy [I, B] [18, 21]. After neoadjuvant chemotherapy (NAC), irradiation is recommended for stage III disease, regardless of response to NAC, and for node-positive disease after NAC [I, A]. For patients presenting with stage II disease (excluding cT3N0) who achieve a pathologic complete response (pCR), radiotherapy could be omitted unless other risk factors [I, B]. [21–23].

Despite these recommendations, benefit of RT for stage I elderly patients has not been proved and should, therefore, be assessed individually [I, A] [24]. Some low-risk patients treated with conservative surgery could be spared whole breast RT and receive partial breast radiation or intraoperative treatment, although less evidence exists to support this approach [25].

## Principles of adjuvant systemic therapy. Genomic profiles in decision making in systemic adjuvant treatment

### Principles of adjuvant systemic therapy

Breast cancer (BC) is a heterogeneous disease, with different subtypes having a distinct biological, molecular, and clinical outcome. Systemic adjuvant treatment is commonly used in early breast cancer with the intention to reduce the rate of locoregional or systemic relapses and death derived from the disease. Treatment decisions are based on clinical (age, comorbidities) and pathologic factors (tumor size, nodal status, grade, hormone receptor (HR) status and HER2 status). Multigenic tests provide information beyond standard clinical and pathologic prognostic factors that can help in making treatment decisions.

### Prognostic gene expression-based assays

Several prognostic gene expression-based assays have been developed to personalize the decision regarding the addition of chemotherapy (CT) in hormone receptor (HR)-positive and HER2-negative BC [26–30].

#### Oncotype DX

The test was initially validated as a prognostic biomarker in tumor samples from several prospective clinical trials (e.g., NSABP-B14 and TransATAC) [30–35]. A score cutpoint of  less than 18 identified low-risk patients at 10 years that could be spared multi-agent chemotherapy, specially in node-negative disease. Less clear was the need of adjuvant chemotherapy in patients with a score of 18 to 31. TAILORx prospective trial in HR+/HER2−/node-negative disease reported that in the intermediate risk group, defined as 11–25, no benefit of adding chemotherapy to endocrine therapy was observed in the overall population at 9 years, although some benefit of chemotherapy was found in women ≤ 50 years of age [29]. An important point is that the vast majority of patients (74%) recruited in TAILORx were clinically low risk (i.e., 63% had tumor sizes 1–2 cm). Indeed, no clear differences in survival outcomes were observed between the intermediate groups (with or without chemotherapy) compared to the low-risk group (i.e., < 11). The RxPONDER prospective clinical trial in 1–3 positive nodes and RS < 25 will shed more light regarding the ability of OncotypeDX RS to predict chemotherapy benefit. Level of evidence: IA for the prediction of adjuvant chemotherapy benefit in patients with clinically low-risk disease and a recurrence score 11–25; IA for the prediction of the risk of distant recurrence at 9 years if treated with adjuvant endocrine therapy-only.

#### Prosigna

This assay classifies a tumor to one of the 4 intrinsic subtypes (Luminal A, Luminal B, HER2-enriched and basal-like) and provides a prognostic 10-year Risk of distant Recurrence (ROR) score [36], which integrates genomic data together with tumor size and nodal status (to determine the risk cutoffs). ROR has now been validated retrospectively in node-negative and node-positive disease in tumor samples from several large adjuvant studies (i.e., TransATAC, ABCSG-08 and Danish cohort) [37–41]. In a recent head-to-head comparison with OncotypeDX RS and EndoPredict in 774 tumor samples from TransATAC, ROR provided a more accurate long-term prognostic information than OncotypeDX, similar to EndoPredict [41, 42]. Prospective validation of the ability of Prosigna to predict adjuvant chemotherapy survival benefit is currently ongoing in large phase III trial in UK (i.e., the OPTIMA trial), in patients ≥ 40 years of age with HR+/HER2−, > 3 cm or node-positive tumors and an ROR below 60 (i.e., low/intermediate). A recent published trial showed the prognostic and predictive value of Prosigna in premenopausal high-risk breast cancer patients treated with cyclophosphamide-based adjuvant chemotherapy [43]. Level of evidence: IB for the prediction of the risk of distant recurrence at 10 years if treated with 5 years of adjuvant endocrine therapy-only; Level of evidence: IB for the prediction of late distant recurrence (years 5–10) if treated with 5 years of adjuvant endocrine therapy-only.

#### Mammaprint

The prognostic value of Mammaprint was first validated in 295 patients with pT1 or pT2 tumors with node-negative or node-positive disease [26, 27]. Since then, other retrospective validations of its prognostic value have been reported. Recently, the primary results of the prospective prognostic validation of Mammaprint have been reported. The MINDACT trial was an international phase III trial designed to evaluate the 5-year distant relapse-free survival (DRFS) in 748 patients, largely HR+/HER2-negative, with a genomic low-risk score and a clinically high-risk score (defined by a modified version of AdjuvantOnline!) when treated without adjuvant chemotherapy. The study met its primary endpoint and this group of patients had a 5-year DRFS of 94.7% (92.5–96.2). However, longer follow-up is needed to determine if this group of patients continues to have an outstanding outcome after 5 years in order to spare them from adjuvant chemotherapy [44]. As a secondary underpowered analysis of the MINDACT trial, Mammaprint did not demonstrate the ability to predict chemotherapy survival benefit. Level of evidence: IA for the prediction of the risk of distant recurrence at 5 years if treated with adjuvant endocrine therapy-only.

#### EndoPredict

The prognostic value of EPclin has been validated retrospectively in tumors samples from 3 large phase III trials (ABCSG-6/ABCSG-8 [45] and TransATAC [46]), where patients did not receive any adjuvant chemotherapy. In addition, the assay has shown ability to predict late distant recurrence [46, 47]. Level of evidence: IB for the prediction of the risk of distant recurrence at 10 years if treated with 5 years of adjuvant endocrine therapy-only. Level of evidence: IB for the prediction of late distant recurrence (years 5 to 10) if treated with 5 years of adjuvant endocrine therapy-only.

## Systemic treatment for luminal-type early stage breast cancer

### Adjuvant endocrine therapy for early stage breast cancer

There is robust evidence that endocrine therapy (ET) improves survival of early stage luminal breast cancer (BC). Adjuvant ET should be offered to any of these patients regardless of age, menopausal status, chemotherapy exposure, expression level of ER or PgR (if any or both are positive defined as ER and/or PR > 1%), and/or Her2 status [I, A]. There are several ET options. The individual choice would be adjusted to menopause status, comorbidity, and the risk of recurrence.

Tamoxifen is the most established adjuvant ET for both premenopausal and postmenopausal women as it reduces the risk of recurrence by 40% in all subgroups [48]. Two large trials (ATLAS and aTTom trial) concluded a higher benefit of continuing Tamoxifen until 10 years, which is recommended for high-risk tumors (at the cost of greater toxicity) [I, B] [49, 50].

Five years of tamoxifen remain the standard treatment for premenopausal women [I, A] [51], but other alternatives should be considered. Adjuvant exemestane plus ovarian function suppression as compared with tamoxifen plus ovarian function suppression or tamoxifen alone significantly reduced the likelihood of distant recurrence in high-risk adjuvant chemotherapy-treated premenopausal breast cancer patients [I, A]. After a median follow-up of 8 years, a survival improvement favoring ovarian suppression plus tamoxifen has been shown [52]. EBCTCG and SOFT trials also support adjuvant tamoxifen plus GnRH analogs as an alternative for high-risk patients not suitable to aromatase inhibitors [II, B] [53].

Several studies explored aromatase inhibitors (AIs) as an initial therapy [54–56], as sequential therapy following 2–3 years of tamoxifen [55–59] or as extended therapy [60, 61] for postmenopausal patients. Different options can be recommended: an AI for 5 years [I, A], tamoxifen for 2 to 3 years followed by an AI to complete 5 years [I, A] or during 5 years [II, B], or tamoxifen for 4.5 to 6 years followed by 2.5 to 5 years of an AI [I, A]. The recently published IDEAL trial did not find differences between 2.5 or 5 years of an AI after 5 years of any ET [62]. The option of 10 years of tamoxifen could be considered in patients with contraindication to AIs or who remain premenopausal.

There is strong evidence to consider the use of bisphosphonates (zoledronic acid 4 mg intravenously every 6 months or clodronate 1.600 mg/d orally) but not an anti-RANK-ligand antibody as additional adjuvant therapy for postmenopausal patients with breast cancer who are candidates for adjuvant systemic therapy. [I, A] [63].

### Adjuvant chemotherapy in hormone receptor-positive early BC

The use of chemotherapy as adjuvant treatment for ER+ Her2-negative disease is recommended for high-risk tumors defined by either clinical or genomic profiling characteristics [I, A], considering: T2 to T4 tumors and/or axillary N2-3 involvement; extensive LVI, high KI67, low ER expression, younger age or premenopausal status; and intermediate to high genomic score. Standard anthracycline and taxane regimens are recommended [90] [I, A].

### Neoadjuvant chemotherapy for luminal breast cancer

Neoadjuvant CT is indicated in LABC [I, A] to reduce the extent of surgery, to treat micrometastases promptly and to monitor the response.

All treatments recommended in the adjuvant setting may also be used in the preoperative setting. If chemotherapy is used, it should be delivered before surgery, without breaks, with the aim to increase the rate of breast-conserving surgery (pCR are infrequent in this setting). A sequential regimen of anthracyclines and taxanes is associated with increased probability of pCR and must be recommended [II, B] [64, 65]. In selected cases of postmenopausal patients with ER+/Her2− disease, neoadjuvant endocrine therapy (NET) during at least 16 weeks is a good option. The available data directly comparing neoadjuvant endocrine therapy (NET) with neoadjuvant chemotherapy are very limited. Some phase II trials and one meta-analysis showed similar response rates, but a significantly lower toxicity with NET [66]. AIs are better to tamoxifen as NET [I, A] [67]. The efficacy evaluation of NET has been performed according to surrogate parameters such as the decrease of the Ki67 levels or the preoperative endocrine prognostic index (PEPI) score [68]. Neoadjuvant ET in premenopausal patients is debatable; an AI with ovarian suppression or tamoxifen could be considered in selected cases in which chemotherapy is not an option [II, D].

In the neoadjuvant setting, chemotherapy is an accepted treatment for tumors with node metastases and for tumors greater than 2 cm who are candidates to mastectomy. We use clinical and pathologic parameters (HR status, grade, Ki-67) to select patients for neoadjuvant chemotherapy (NCT), though there is no agreement about the cutoff point of Ki-67 and about the accuracy of this marker to predict chemotherapy response. Different genetic signatures have been evaluated in core needle biopsy before neoadjuvant therapy, as good predictors of response to neoadjuvant therapy, especially PAM50 ROR score [69], although this approach is currently considered experimental.

## Systemic treatment for early stage her2-positive breast cancer

### Adjuvant treatment for HER2-positive disease [70–77]

The addition of trastuzumab to chemotherapy has dramatically improved prognosis for early stage HER2-positive breast cancer patients. The benefits of trastuzumab are independent of age, tumor size, nodal and HR status. Adjuvant trastuzumab is recommended in node-positive and node-negative tumors with a tumor size > 1 cm [I, A]. No level I evidence exists regarding the use of trastuzumab in node-negative and tumors ≤ 1 cm, although it might be considered in most patients with tumor size 0.5–1.0 cm [II, B]. While trastuzumab added sequentially after chemotherapy has demonstrated activity, results from the NCCTG N9831 study and a meta-analysis suggest better outcomes when trastuzumab is given concurrently with taxane-based treatment. Thus, AC or EC for 4 cycles followed by 3 months of paclitaxel (P) or docetaxel (D or T) both in combination with trastuzumab (AC/EC → P/D + H) or docetaxel, carboplatin and trastuzumab (TCH) are preferred regimens [I, A]. It is important to consider cardiac risk factors when determining the appropriate chemotherapy backbone to administer with trastuzumab. In small, node-negative tumors (stage-I), although not all patients require adjuvant trastuzumab-based chemotherapy (particularly those with pT1aN0), a less intense chemotherapy regimen such as single-agent paclitaxel and trastuzumab for 12 weeks followed by single-agent trastuzumab to complete one year provides excellent outcomes [II, B]. To date, 12-month duration of trastuzumab remains the standard of care in most situations [I, A]. However, in patients with cardiac toxicity or at very high risk of cardiac toxicity, a shorter course (i.e., 6 months duration) can be considered. Trastuzumab may also be safely combined with either radiotherapy or endocrine therapy.

While there have been many efforts to improve outcomes even further, the addition of novel anti-HER2 therapies to trastuzumab, such as pertuzumab or neratinib, has led to significant, but modest improvements in DFS, and no impact on OS has yet been reported. Balancing risks and benefits is critical when evaluating adjuvant dual blockade with pertuzumab plus trastuzumab or extended HER2 inhibition with neratinib following 1-year of trastuzumab. Up to 18 cycles of adjuvant pertuzumab in combination with trastuzumab-based chemotherapy showed a 19% relative reduction in invasive disease-free survival (iDFS) at 3 years in the large phase III APHINITY trial. When stratification factors were evaluated, patients with high-risk features such as node positive or HR negative derived a significant iDFS, whereas patients with node negative or HR+ disease did not. Although longer follow-up is needed, EMA states that the available data do not allow concluding for a positive benefit-risk ratio in the overall population. However, EMA considers that benefit is clearly shown in the high-risk population (HR-negative or node-positive) [I, B] for a total duration of 18 cycles of dual blockade, regardless if it was initiated in the adjuvant or the neoadjuvant setting.

The addition of 1 year of adjuvant neratinib improved iDFS in patients with HER2-positive early breast cancer after 1 year of trastuzumab, as demonstrated in the phase III EXTENET trial. However, the benefit was higher in patients with HR-positive and node-positive disease, at the expense of increased diarrhea [I, B]. Recently, neratinib has been approved by EMA, which restricted its use to HR+ disease. However, there are no data on the added benefit of neratinib in patients who also received pertuzumab.

### Neoadjuvant treatment for HER2-positive disease [78–81]

In the neoadjuvant setting, trastuzumab in combination with chemotherapy (taxane and anthracycline based) has been the standard treatment for HER2-positive tumors with nodal involvement or tumor size > 2 cm. Two phase II trials evaluated the addition of pertuzumab to trastuzumab-based chemotherapy, demonstrating higher rates of pCR. These data, along with its efficacy in terms of OS in the metastatic setting, led to the accelerated approval of this combination in the neoadjuvant scenario. Dual blockade with trastuzumab and pertuzumab and chemotherapy (anthracycline plus taxane based, or anthracycline-free regimens) should be considered for the treatment of HER2-positive breast cancer patients who meet criteria for neoadjuvant treatment (i.e., > 2 cm tumor size or node positive). According to EMA and FDA, treatment with dual HER2 blockade can be continued after surgery for up to 18 cycles [I, B]. At this point, the type of pathological response at surgery should not guide the duration of trastuzumab or pertuzumab since there are no data supporting one strategy or another. Finally, the addition of lapatinib to trastuzumab and chemotherapy has not consistently improved pCR rates and long-term outcome. To date, this combination cannot be recommended for the treatment of patients with early stage HER2-positive breast cancer [I,E].

## Systemic therapy for early stage triple-negative breast cancer

### Adjuvant treatment for triple-negative disease

Triple-negative breast cancer (TNBC) is a heterogeneous disease comprising approximately 15% of all breast cancers. With the exception of medullary, adenoid cystic, and apocrine carcinomas that have a better outcome, TNBCs have generally an aggressive behavior.

Conventional chemotherapy remains the mainstay of adjuvant systemic treatment for most patients with early TNBC. Adjuvant chemotherapy should include an anthracycline and a taxane [I, B], although the regimen docetaxel-cyclophosphamide might be considered in patients with a high risk for cardiac toxicity. Nevertheless, women with T1a/bN0 tumors have an excellent prognosis without chemotherapy [82]. No adjuvant chemotherapy is recommended in tumors equal or less than 0.5 cm (pT1a), and for 0.6–1 cm tumors, it has to be discussed and balanced [III, B]. No robust, prospective randomised data exist on the use of platinum compounds in the adjuvant setting, either in unselected triple-negative tumors or BRCA 1/2 mutation carriers.

### Neoadjuvant treatment for triple-negative disease

Neoadjuvant therapy in TNBC leads to pCR rates of 30–40%, which has been associated with an excellent prognosis [65]. It is recommended that a sequential regimen of anthracyclines and taxanes is used for the vast majority of patients [I, B]. An improved pCR was observed with Nab-paclitaxel compared to solvent-based weekly paclitaxel (43 vs 34%) in a head to head phase III neoadjuvant trial. This effect was seen in all subgroups, especially in TNBC patients [83].

Platinum-based neoadjuvant chemotherapy significantly increased pCR from 37.0% to 52.1% (OR 1.96, 95% CI 1.46–2.62, *P* < 0.001), with significant higher risk of grade 3 and 4 hematological AEs [I, A]. In the 96 BRCA-mutated patients included in two randomized controlled trials, the addition of carboplatin was not associated with significantly increased pCR rate [84]. The effect of those compounds on long-term outcomes is unknown [I, B].

Addition of 6–8 cycles of adjuvant capecitabine therapy among patients who had residual invasive disease on pathological testing after anthracycline and taxane-based neoadjuvant chemotherapy was safe and effective in prolonging DFS and OS in the randomized phase III CREATE-X trial [85]. Based on those results and a meta-analysis [86], adjuvant capecitabine can be considered in high-risk TNBC patients with residual invasive disease at surgery following standard neoadjuvant chemotherapy [I, B] This treatment, though, must be balanced against potential toxicities.

Recommendation: sequential regimens of anthracyclines followed by taxanes are the standard treatment. Platinum-based neoadjuvant chemotherapy may be considered an option in TNBC patients [I, A].

## Follow-up

Follow-up after surgery and adjuvant treatment of breast cancer patients is widely reported in guidelines worldwide. Surveillance for breast cancer recurrence, screening for second primary cancers, assessment and management of physical and psychosocial long-term and late effects of breast cancer and its treatment, and health promotion are encouragingly recommended [I, A] [87]. Healthy lifestyles, such as physical exercise and avoidance of obesity, are recommended to prevent tumor recurrence [II, A] [88].

Despite the fact that no randomized data exist to support any particular follow-up sequence or protocol, balancing patient needs and follow-up costs, we recommend regular visits every 3–6 months in the first 2 years, every 6 months from years 3–5 and annually thereafter [III, A]. Every visit should include a thorough history, eliciting of symptoms and a physical examination. Annual ipsilateral (after BCT) and/or a contralateral mammography with ultrasound is recommended [II, A]. An MRI of the breast may be indicated for young patients, especially in cases of dense breast tissue and genetic or familial predispositions [89].

## Abbreviations

ET: endocrine therapy
TAILORx: Trial Assigning Individualized Options for Treatment
MINDACT: Microarray in Node-Negative and 1 to 3 Positive lymph Node Disease may Avoid Chemotherapy EORTC 10041/BIG 3-04 study
DCIS: ductal carcinoma in situ
